# Light Dulls and Darkens Bird Eggs

**DOI:** 10.1371/journal.pone.0116112

**Published:** 2014-12-26

**Authors:** Johanna Y. Navarro, David C. Lahti

**Affiliations:** Department of Biology, Queens College, City University of New York, Flushing, New York, United States of America; Hungarian Academy of Sciences, Hungary

## Abstract

Although egg color is generally consistent within individual birds and robust to environmental variation, recent evidence suggests a degree of susceptibility to environmental perturbation or modulation of egg color. Most of this variation manifests via the physiology of the laying female, but some direct impacts of the environment on laid eggs have also been discovered. Here we test whether light changes bird egg color and we quantify its effect, by subjecting variable blue-green eggs of Rüppell's weaver (*Ploceus galbula*) to a broad-spectrum light source under laboratory conditions, and measuring egg reflectance every few hours. Eggshells gradually decreased in reflectance across the entire measured wavelength spectrum of 250–800 nm. Reflectance peaks were disproportionately affected, such that the height decreased of both the blue-green peak and the smaller UV peak typical of blue-green eggs. The reflectance of lighter eggs was affected slightly more than that of darker eggs. These changes are similar to previous results for changes over long periods of time in darkness, suggesting that light might hasten the same process of pigment degradation that proceeds even without light. Comparison between the experimental light source and both sunlight and typical artificial lighting situations raises the possibility that significant color change might occur during incubation in some birds, but indicates that eggshell illumination in museums for short periods of study is unlikely to affect their color to a detectable extent. Additional research should be performed on eggs of other species and in other light environments, with an eye to an eventual generalized model of the effect of light on eggshell color.

## Introduction

In 1897, experimental evidence corroborated the impressions of bird egg collectors and museum curators that the colors of bird eggs “are not very fast to light” [1]. This initial discovery that egg coloration changes following light exposure was thoroughly overshadowed in the subsequent century's research on egg color, which was dominated by the discoveries that it is consistent within an individual bird and has a strong genetic basis. Punnett's [2] breeding experiments demonstrated that egg color differences in Chilean fowl (*Gallus gallus* Araucana) are genetically regulated, and that blue egg color is dominant to non-blue and can combine with gradients of brown through breeding to create a variety of egg colors. Work in this vein has continued to specify the inherited component of egg color, e.g., [3]–[6]. Recent research on the genetics of blueness in chicken eggs has culminated in the discovery of a retroviral insertion (*EAV-HP*) that strongly influences this trait [7]. Concordant with these and other studies of the genetic bases for bird egg color differences, egg color has generally been shown to be robust to environmental variation [6], [8]–[12], licensing claims of evolutionary change from population comparisons [13]. Nevertheless, a recent increase in the precision of color measurement [14] and the proliferation of hypotheses for the functions of bird egg color [15]–[18] have accompanied a surge of interest in the role of environmental and developmental features in modulating or disrupting egg color. This investigation has met with some success: for instance, environmental pollutants have recently been implicated in egg color variation [19], [20]; and dietary manipulation has elicited changes in egg color [21], [22]. Moreover, changes in egg color have been observed to relate to bird age [23], female condition [24], and laying order [21], [25]. Accumulating instances of small-scale spatial and temporal variation in egg color, especially when correlated with environmental gradients, have also been suggestive [15], [25]–[28]. In all of these cases, the changes in egg color are considered to be modulated by the physiology of the mother, and therefore occur before the egg is laid.

Less researched is the extent to which environmental features directly impact egg color after laying. Some superficial examples of this are well known, such as staining and the wearing off of bloom [29]. Field researchers typically notice a change in the translucence and perhaps color of eggs after laying, an effect corroborated by the finding of Moreno et al. [30] that the proportional contribution of blue-green wavelengths to eggshell reflectance declines after laying and through the incubation period in the pied flycatcher (*Ficedula hypoleuca*). Somewhat better investigated is the change in egg color while in museum collections, following analogous work on plumage [31], [32]. Specifically, the proportional contribution of blue-green wavelengths to eggshell reflectance was lower in museum specimens than in freshly laid eggs of the song thrush (*Turdus philomelos*), and older egg specimens were less blue-green (according to the same metric) than more recent specimens [33]. Moreover, in both this species and in the blackbird (*Turdus merula*), eggs reserved for five years in darkness and measured again were less blue-green as well as lower in reflectance overall [34]. Similar results were found in pied flycatcher eggs after 6 months, and again after 23 months, of dark storage [30].

Possible change in egg color with time, particularly in museum specimens, has sometimes been referred to as light-induced fading. There are two problems with this hypothesis. First, the role of light is open to question given the changes that were observed in darkness by Cassey et al. [34] and Moreno et al. [30]. Second, the only quantitative evidence of egg color change over time is a *decrease*, rather than an increase, in reflectance [30], [33], [34], which is opposite of the usual meaning of fading as bleaching, i.e., an increase in reflectance, as occurs through photodegradation in a variety of dyed objects such as fabrics [35], and in coral through a combination of biological and physical mechanisms [36]. On the other hand, fading could also be taken to mean a loss of chroma [37], i.e., dulling, which is more consistent with past results from eggshells.

Porphyrins, the class of pigments responsible for egg color, are known to photodegrade [38], [39]. Moreover, some influence of light on egg color can readily be observed in permanent eggshell displays in bright places; this danger is widely accepted among museum curators, hence the longstanding practice of keeping egg specimens in darkness [1], [40]. Also, in the most spectacular instance of natural change in egg color, emu (*Dromaius novaehollandiae*) eggs can be as dark as the skin of an avocado at an early stage, but will fade nearly to white upon exposure to sun, even over a typical incubation period [29].

Here we expose bird eggshells to a known intensity and color of broad-spectrum light in a controlled environment to assess the effect of light on their reflectance. Generalities can best be drawn if experimental eggs vary, but many other things about eggs besides color vary across species [41]. We solved this problem by testing eggs of a species that lays variably colored eggs: Rüppell's weaver (*Ploceus galbula*). Rüppell's weaver egg colors are reported as ranging from a medium blue to white, usually densely spotted with dark red speckles or, less frequently, with tan speckles [42]. Here we ask whether and how light affects the color of these eggshells, including in what area of the reflectance spectrum changes occur, whether chroma and brightness are both affected, whether initial reflectance matters, and how the effects relate to duration of exposure.

## Methods

**Figure 1:**
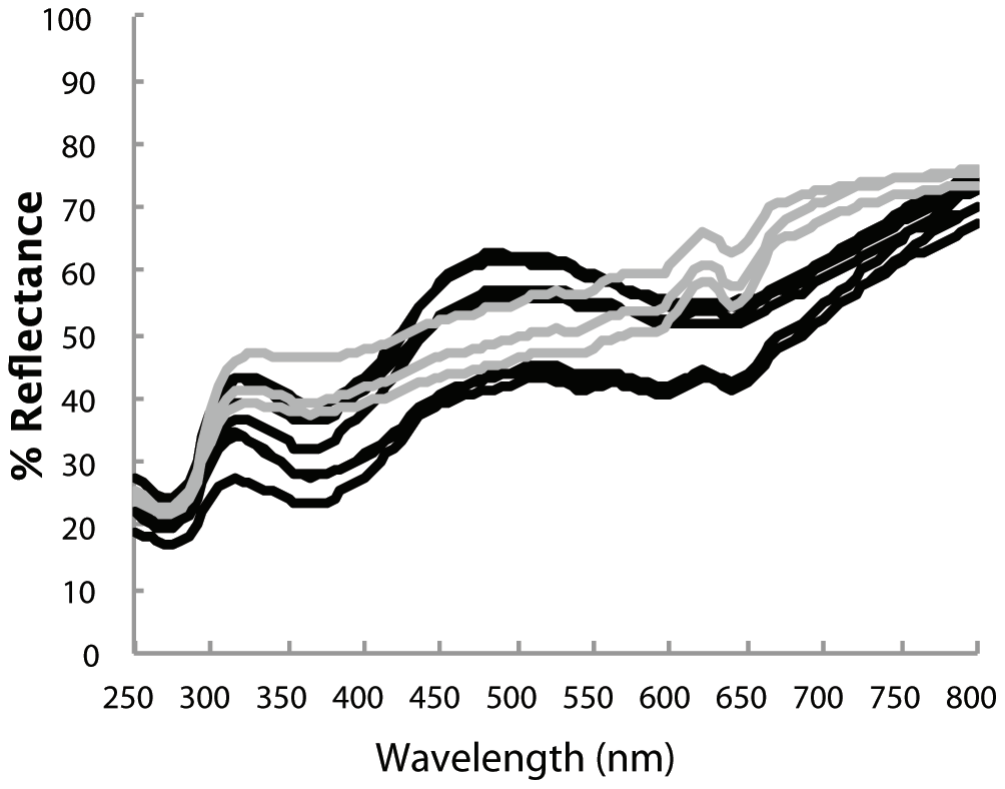
Ground color reflectance spectra of the ten Rüppell's weaver eggshells used in this study. Most (7) eggs ranged between very light and light blue-green (peak around 500 nm), but in three cases indistinct tan spots clouded the ground and no blue-green peak was evident (gray lines).

Eggs of Rüppell's weaver (*Ploceus galbula*) were collected by DCL less than two days after laying in August 2010 at Awash National Park, Ethiopia (8°53.150′ N, 40°02.147′ E). Each egg was from a different nest. All eggs appeared to be light to very light blue-green in ground color, with spots ranging from tan to rust, sometimes so fine or indistinct as to slightly obscure the ground color. Rüppell's weaver is endemic to the Horn of Africa and the southern Arabian peninsula and is not well studied [42], [43]; however, no eggs were taken specifically for the present study. These eggs were collected in order to quantify egg appearance after egg replacement experiments, during which time the weavers promptly renested. On the day of collecting, each eggshell was emptied of its contents and stored in a dark box after drying and until the present study began. Ten eggs were selected for the study based on visual assessment to maximize the range of variation in ground color. See Fig. 1 for reflectance spectra of all study eggs. DCL conducted all studies in accordance with permitting regulations in both the U.S.A. and Ethiopia, including permission from the Ethiopian Wildlife Conservation Authority that has jurisdiction over Awash National Park, and according to the recommendations in the Guide for the Care and Use of Laboratory Animals of the National Institutes of Health. The original protocol including the collection and use of eggs was approved by the Committee on the Ethics of Animal Experiments of Queens College, City University of New York (QC IACUC Permit Number 144); no additional IACUC approval was necessary for the subsequent use of eggshells as described in the present study.

The treatment consisted of placing eggs in shallow depressions beneath a light source in a laboratory such that the side of each egg faced towards the light for a total of 108 hours. At specified intervals during this period each egg was removed from the light in order to measure its reflectance: at the start, and then after 4, 12, 20, 28, 36, 60, 84, and 108 hours of light exposure. Reflectance was measured with an Ocean Optics USB2000 UV-VIS spectrophotometer, associated Spectrasuite software (Ocean Optics, Dunedin, Florida, USA), a 200 Hz pulsed xenon light source (Ocean Optics PX-2), and a 400 µm reflection probe (Ocean Optics R400-7), held perpendicular to the egg at 7 mm distance, under an opaque cloth. Integration time was set at 80 ms. Reflectance measurements were standardized with a diffuse polytetrafluoroethylene (PFTE) tile that reflects >98% of light over all sampled wavelengths (Ocean Optics WS-1). A rubber stopper was placed over the probe to maintain a consistent angle and distance from the probe to the eggshell. One of us (JYN) made all reflectance measurements, focusing on the center of the eggshell lengthwise, making an effort to avoid spots in order to measure ground color. A photograph was taken of the face of the egg that was exposed to the light, and was consulted in order to ensure that the same area of the egg was measured at every interval, and that the egg was replaced with the same face towards the light after each measurement. Every reflectance measurement was the average of three reads within a 1 cm^2^ area of the egg (repeatability 0.99). To detect any change in the spectrophotometer or non-light related changes in egg color over the study period, four additional weaver eggs were measured at each interval but not subjected to the light treatment, remaining instead in a dark box for the duration except when measured for reflectance; no change in the reflectance spectra of these eggs was observed. We gathered reflectance between 250 and 800 nm, a range extending well beyond the bird or human visible wavelengths, allowing us to assess changes in ultraviolet (UV) and infrared (IR) reflectance of the eggshells as a result of light exposure.

We used a 26.67 cm long, 105 watt compact fluorescent bulb (CFS32, US Way Lighting), which produces 6720 lumens at a color temperature matching that of mean daylight at 6500 K [44]. By comparison, the color temperature of typical incandescent light bulbs is no higher than 3300 K, and that of ordinary compact fluorescent light bulbs is about 5000 K. We suspended this bulb within a white cone reflector such that the base of the bulb was 29.2 cm above the eggs. This setup resulted in the eggs being exposed to 23.3 W/m^2^ of light between 250–800 nm, as determined by absolute irradiance measurements taken with the above spectrophotometer, calibrated to a light source of known power (Ocean Optics DH2000-CAL, deuterium tungsten-halogen), and using a 450 µm optical fiber, with a 3.9 mm PFTE cosine corrector (Ocean Optics CC-3-UV-T). Despite the broad spectrum of the bulb's lighting, 96.1% (22.4 W/m^2^) of its power fell (not surprisingly) in the human-visible wavelength range (400-700 nm). Light exposure is compared to selected natural and artificial environments in Table 1, as measured with the same equipment.

**Table 1:** Comparison of the experimental light treatment in this study with natural and artificial light environments relevant to field and museum researchers/curators.

	250–800 nm (total)		250–400 nm (UV)		400–700 nm (VIS)		700–800 nm (IR)	
	W/m^2^	photons/cm^2^/s	W/m^2^	photons/cm^2^/s	W/m^2^	photons/cm^2^/s	W/m^2^	photons/cm^2^/s
This study (see text)	23.3	6.14×10^15^	0.542 (2.3%)	9.66×10^13^ (1.6%)	22.4 (96.1%)	5.92×10^15^ (96.4%)	0.348 (1.5%)	1.25×10^14^ (2.0%)
Museum/Lab (fluorescent: single linear tube 2m above table)	1.17	3.24×10^14^	0.0153 (1.3%)	2.86×10^12^ (0.9%)	1.13 (96.2%)	3.11×10^14^ (96.0%)	0.0288 (2.5%)	1.04×10^13^ (3.2%)
Museum/Lab (incandescent: 65W recessed floodlights 2m above table)	0.834	2.85×10^14^	0.006 (0.7%)	1.03×10^12^ (0.4%)	0.433 (51.9%)	1.33×10^14^ (46.7%)	0.397 (47.6%)	1.51×10^14^ (53.0%)
Bright desk (fluorescent: two 15W linear tubes 0.3m above desk)	7.51	2.03×10^15^	0.264 (3.5%)	4.81×10^13^ (2.4%)	7.11 (94.7%)	1.93×10^15^ (95.1%)	0.132 (1.8%)	4.90×10^13^ (2.4%)
Bright desk (incandescent: 60W bulb in a hemispherical metal shade 0.3m above desk)	14.8	4.98 ×10^15^	0.0257 (0.2%)	6.90×10^12^ (0.1%)	8.27 (55.9%)	2.50×10^15^ (50.2%)	6.53 (44.1%)	2.47×10^15^ (49.6%)
Direct sun, 1600 hrs, 27 June, sun angle 50°, New York (41.4°N)	392	1.14×10^17^	21.3 (5.4%)	4.27×10^15^ (3.8%)	297 (75.8%)	8.18×10^16^ (71.8%)	74.0 (18.9%)	2.79×10^16^ (24.5%)

Watts per square meter are given as a typical measure of irradiance. Photons per square centimeter per second are also given because, for a given region of the spectrum, these might bear a linear relationship with change in egg color over time. These two metrics of irradiance contribute different percentages of their respective totals because the power (W/m^2^) of a photon varies according to wavelength.

**Figure 2:**
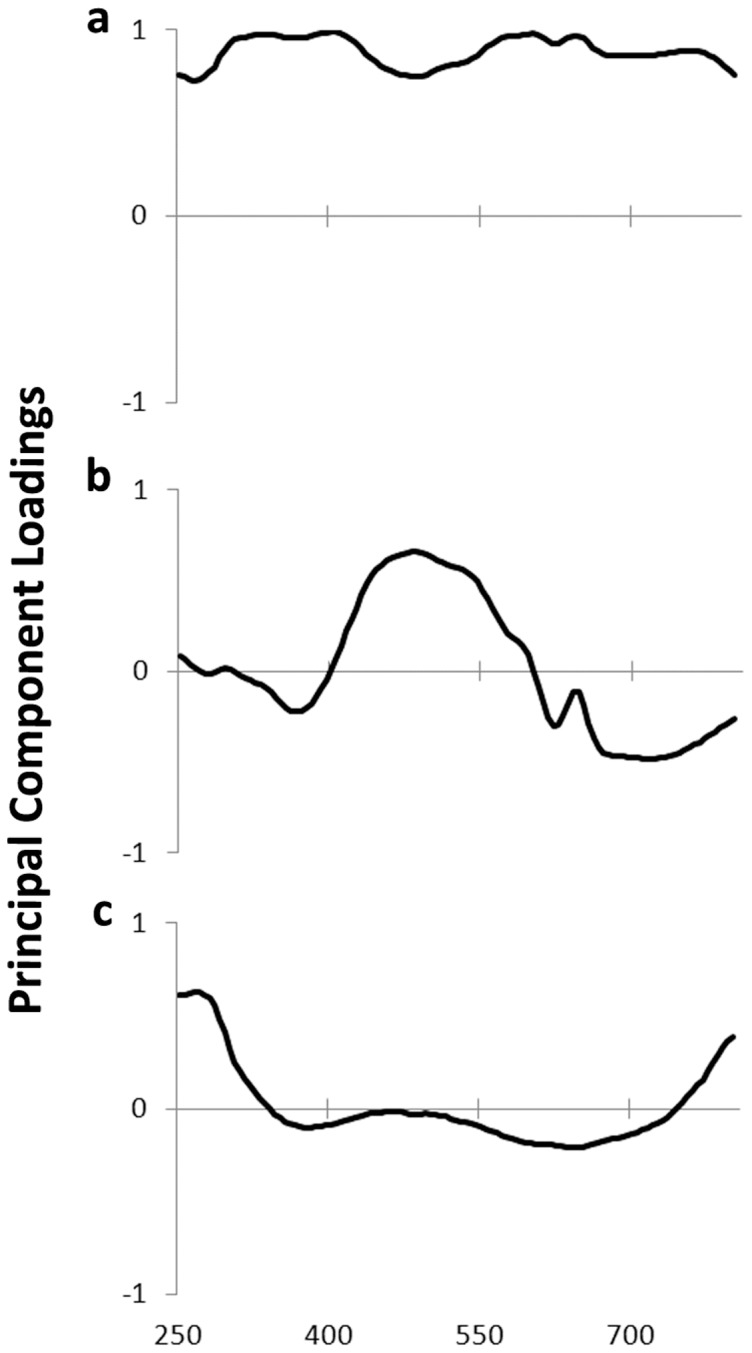
Principal component loading graphs (a: PC1, b: PC2, c: PC3), representing the relative extents to which areas of the reflectance spectrum contribute to the respective PCs. See Table 2 for changes in these PCs upon exposure to light.

We analyzed the change in reflectance over time of light exposure in two different ways: (1) through two steps of statistical data reduction, and (2) through a direct analysis of spectral shape. We used SYSTAT 10 (SPSS, 2000) for all analyses. In the first analysis, we reduced spectral data across wavelengths with principal components analysis and then across time by regression. Rüppell's weaver eggs generally have two reflectance peaks, one in the UVB region (centering around 310 nm) and one blue-green (centering around 500 nm) (Fig. 1). The unrotated component loadings were largely interpretable as brightness, blue-green chroma, and UVB-C/IR (Fig. 2). These three components explained 78.6, 13.9, and 4.9 percent of the variance, respectively. We calculated the resulting component scores for each egg across all reflectance measurements during light exposure. We then ran a linear regression (LR) for each component per egg, regressing all nine values of the component against time, in order to reduce the eight temporal transitions to a single slope per egg. We calculated the 95% confidence interval around these ten slopes; if they did not include zero, we rejected the null hypothesis of no change in the component over time. In the second analysis, we derived three variables directly from the reflectance spectra: the average reflectance across 250–800 nm (brightness); the height of the blue-green peak, calculated as R500 – (R400 + R650)/2 (blue-green chroma [17]); and the height of the UV peak, calculated as R310 – (R270 + R350)/2. The first term in each relation is the typical location of the peak (local maximum reflectance); and the second term is the mean of the two typical locations of the troughs (local minimum reflectance) flanking the peak (see Fig. 1). We estimated peak height using these standard wavelengths because they approximate the inflection points (±10 nm) and also facilitate repeatability [17], [45]. We excluded three eggs from the blue-green analysis because tan spots occluded the ground color and so those eggs did not have the requisite peak. We then conducted a repeated-measures ANOVA across the time of light exposure. For graphical representation we standardized the values of these three variables by subtracting all values during light exposure from the initial value, thus portraying change from the starting condition. Finally, in order to address the question of whether any light-induced change in reflectance differs according to how bright the eggs are, we regressed the total change in brightness against the initial brightness of the eggs. All interpretations are based on p = 0.05 as the threshold for significance.

## Results

**Figure 3:**
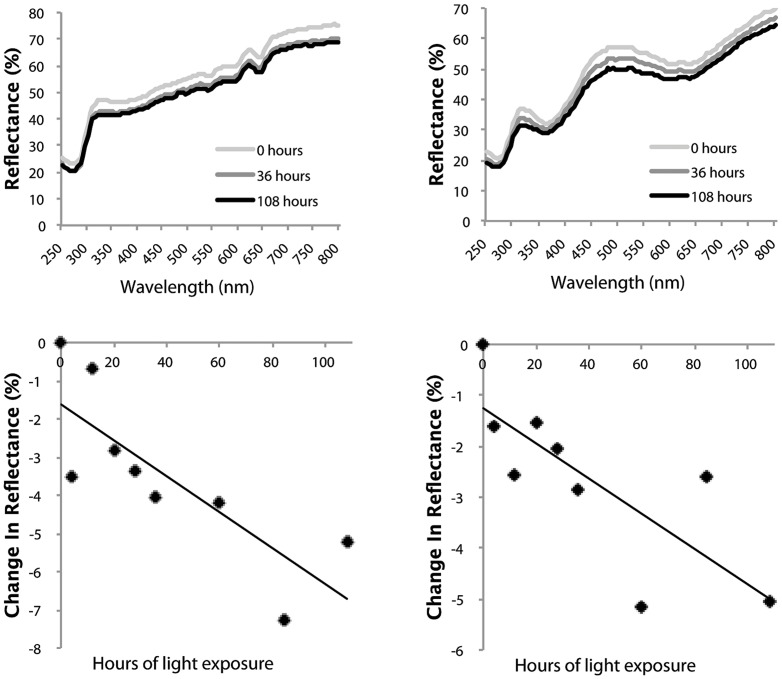
Decrease and flattening of spectral reflectance of two representative study eggs following exposure to broad-spectrum light. The top panels show the reflectance measurements of each egg at the start of the study and following 36 and 108 hours of exposure to broad-spectrum light. The bottom panels display the change in average reflectance across 250–800 nm throughout the treatment.

**Figure 4:**
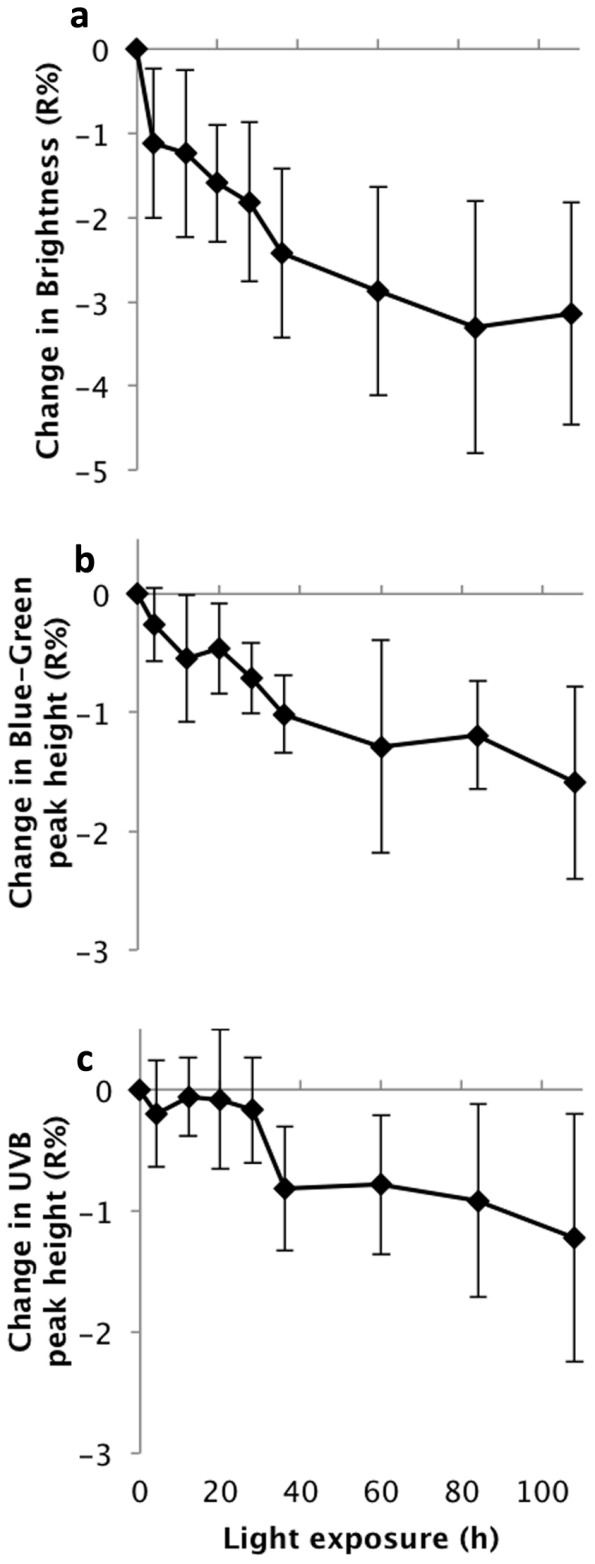
Dulling of eggshell color following exposure to broad-spectrum light. **(a)**. Decrease in average spectral reflectance of all ten study eggs across 250–800 nm. **(b)**. Decrease in blue-green peak height (height of the 550 nm reflectance peak above the average of 400 nm and 650 nm) calculated for the seven study eggs that exhibited such a peak. **(c)**. Decrease in UVB peak height (height of 310 nm reflectance peak above the average of 270 nm and 350 nm), calculated for all ten study eggs.

**Figure 5:**
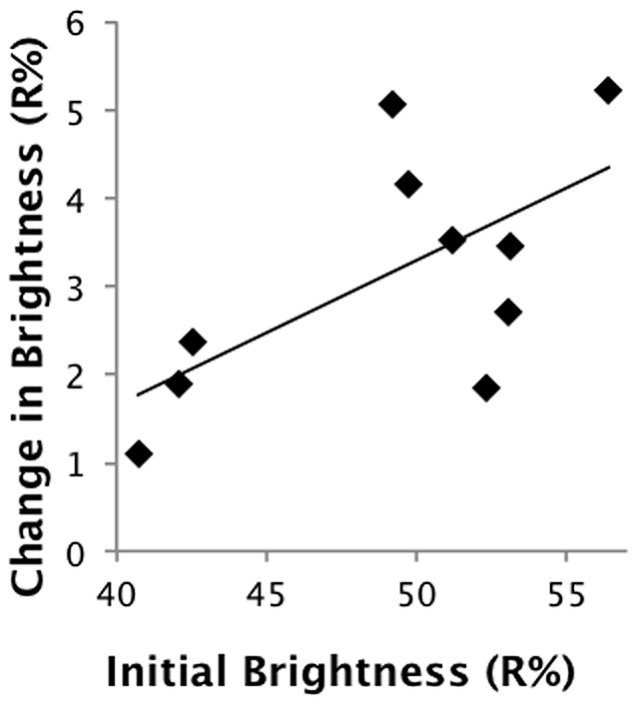
Change in brightness of eggs as a function of the initial brightness (average reflectance across the spectrum) of the egg. The brighter the egg, the greater effect light tended to have.

All three principal components—interpretable largely as brightness, blue-greenness, and UVB-C/IR respectively—had negative slopes over the period of light exposure, and the associated confidence intervals did not cross zero (Table 2). This effect can likewise be seen by direct inspection of the reflectance spectra and in the changes through time of each egg without exception (see Fig. 3 for two examples). Analyzing all ten eggs together, repeated measures ANOVAs were highly significant across the eight time periods for all three direct spectral variables: incident light caused a decrease on average reflectance (F_8, 72_  =  11.81, p<0.0001), a decrease in the height of the blue-green peak (of the seven blue-green eggs) relative to the surrounding areas of the spectrum (F_8, 48_  =  11.50, p<0.0001), and the height of the UVB peak (F_8, 72_  =  11.39, p<0.0001) (Fig. 4). Eggs with a higher initial reflectance across the spectrum tended to decrease in average reflectance to a greater extent than darker (lower reflectance) eggs (LR: R^2^ = 0.41, p = 0.048) (Fig. 5). Neither the patterns of residuals from the egg-specific linear regressions, nor the patterns of data points for the individual eggs (Fig. 3) or the aggregated data (Fig. 4), strongly indicate nonlinearity over this duration of exposure, although the initial change in brightness after four hours did tend to be larger than subsequent changes. A rigorous test of linearity would require finer-scaled and evenly spaced intervals of exposure.

**Table 2:** Effects of broad-spectrum (visible) light on principal components (PCs) of eggshell reflectance.

	Slope mean (SD)	Slope 95% CI
Brightness (PC1)	−0.0058 (0.0021)	−0.0043 to −0.0073
Blue-greenness (PC2)	−0.0017 (0.0018)	−0.0004 to −0.0030
UVB-C/IR (PC3)	−0.0103 (0.0033)	−0.0079 to −0.0127

See Fig. 2 for the relationship between PCs and reflectance spectra. Slope data are from egg-specific linear regressions of each PC on exposure time in hours. A negative slope indicates that the color variable decreases with time when exposed to light. Small magnitudes of the slopes indicate that the changes in response to the light treatment are dwarfed by the variation among eggs (the Rüppell's weaver has variably colored eggs).

## Discussion

Exposure to broad-spectrum visible light caused eggshells of the Rüppell's weaver gradually to lose reflectance across the entire measured spectrum of 250–800 nm. Reflectance peaks were disproportionately affected, in this case resulting in a decrease in blue-green chroma and in the smaller UV peak. Lighter eggs were affected slightly more than darker eggs. Analyzing the data by data reduction (PCA) followed by the calculation of regression slopes for each egg produced similar results to the direct analysis of spectral features, with the exception that the change in the component that loaded heavily on UV as judged from the PC slope was greater than the change in UV as judged from direct analysis of the spectral peak, likely due to the inclusion of infrared (IR) changes in that PC (see loadings in Fig. 2). Because PCs can be somewhat less tractable in this way, we find the direct analysis of spectra to be more immediately interpretable when such an analysis is feasible. The decrease in reflectance across the spectrum amounts to a slight darkening of eggs following exposure to light. The decreases in the blue-green peak reflectance can be interpreted as dulling, or loss of color intensity. The UV peak extends partly into the bird-visible range, and thus its decrease could be considered partly as dulling as well. These effects also extend into the nonvisible regions of the UV and IR. As with any nearly opaque substance, any radiation that is unreflected will be mostly absorbed, with some smaller proportion transmitted into the egg.

These results are consistent with the qualitative conclusions of Paterson [1], namely that blue-green eggs became redder and duller following light exposure. He also noted that darker (brownish) eggs did not change as much or at all. The results of the present study can only be interpreted as relevant to blue-green eggs (i.e., eggs predominantly colored with biliverdin) until similar experiments are performed on eggs of other colors. The decrease in reflectance we observed is also consistent with recent studies of changes in blue-green eggs over time in darkness [3030,33,34]. Those studies interpreted chroma differently than in this study, as proportional contribution of a certain area of the spectrum to total reflectance, resulting in an automatic change in chroma for any change in brightness. Still, their results do show similar changes in chroma as interpreted in this study as peak height. Remarkably, then, broadly similar changes occur to egg colors over long periods of time in complete darkness, as occur over short periods of time exposed to light. This similarity leads us to speculate that a single process, for instance the oxidative degradation of pigment, might be occurring, some reaction that although not requiring light is hastened by it.

We expect that the changes we observed are caused or catalyzed by photon bombardment of the shell surface, such that additional photons at the same wavelengths will have an additive effect on pigment photodegradation. Our study eggs were reserved in darkness for four years before this study; presumably blue-green eggs tested after a much longer time will still respond to light in a similar way. If this is the case, the results of the present study together with data such as that in Table 1 can be used to generate provisional estimates of the change in spectral reflectance over time of exposure, aside from the rate at which brightness and chroma decrease without light. The experimental lamp in this study resulted in 1% decrease in reflectance of our study eggs in about 30 hours on average, plus 0.5% decrease in the blue-green peak height and a 0.2% decrease in the UVB peak height over the same time period. If, as indicated in Table 1, fluorescent desk lighting is approximately a third of the power of the study lamp, thus emitting a third of the photons over roughly the same wavelength range, then we might expect one third of the effect. This translates into a 0.011% decrease in reflectance for every hour a blue-green egg in a collection is out of the specimen drawer on a lit table, and an additional half that decrease in blue-green chroma, just from the effect of light. However, this prediction treats ordinary fluorescent light as equivalent to the broad-spectrum, high color temperature bulb used in this study, which might not be the case. Similar rough predictions could be derived regarding the expected decrease in reflectance during incubation in nature, if we knew the dynamics of solar irradiance and duration of incidence on the eggs. Likewise such predictions might be skewed if the sun's UV and IR radiation contributes to the effect on egg color (the study lamp's radiation attenuated rapidly in the UV and IR regions compared to sunlight (Table 1)). More helpful data regarding the effect of solar radiation would come from direct studies of eggshells in sunlight, although variation in sunlight would necessitate regular irradiance measurements throughout the experimental period. Biological relevance would additionally require study of incident light on birds' eggs in natural nests. In addition, previously collected eggshells, refilled, placed into active nests, and measured periodically along with the natural eggs for reflectance changes, could distinguish between changes due to solar radiation and those due to maturation following laying.

Just as different sources of light might have various effects on eggshell color, different species' eggs might not respond the same way, either because of the initial color of the eggs or because of other eggshell characteristics. The ranges of hue, chroma, and brightness of the eggs in this study were limited, but still variable enough to show that different colors change at different rates. Similar research should be conducted on the eggs of other species, and under other lighting conditions, to determine the importance of this variation. Eventually, since both light and eggshells vary in a limited number of quantitative variables, we should be able to model eggshell reflectance changes in a general way that can accommodate differences among species and light environments.

Our results contribute to the growing realization that despite the overall consistency of egg colors across a broad range of environmental and dietary circumstances, some environmental factors do cause egg color to change. We cannot speculate on the possible changes to typical blue-green eggs over incubation specifically due to light because incident light on natural eggs will vary widely due to parental behavior, and nest and habitat characteristics. Still, the fact that direct sunlight is an order of magnitude brighter than our study lamp (Table 1) raises the possibility that the effect of light on blue-green eggs in nature could be important. The implications for museum curation and collections-based research are slight, however: light does dechromatize and darken bird eggshells, at least of certain colors, but it does so gradually. A blue-green egg could be subjected to many hours of study by many researchers in a typical museum light environment before the change in reflectance of a blue-green egg exceeded the measurement error of the most skillful spectrophotometrist.

## References

[1] Paterson D (1897) The effect of sunlight on the tints of birds' eggs. Nature 56:11.

[2] Punnett RC (1933) Genetic studies in poultry, IX. The blue egg. Journal of Genetics 27:465–470. 10.1007/BF02986632 18905081

[3] Punnett RC, Bailey PG (1920) Genetic studies in poultry, II. Inheritance of colour and broodiness. Journal of Genetics 10:277–292.

[4] Hutt FB (1949) Genetics of the Fowl. New York: McGraw-Hill.

[5] Francesch A, Estany J, Alfonso L, Iglesias M (1997) Genetic parameters for egg number, egg weight, and eggshell color in three Catalan poultry breeds. Poultry Science 76:1627–1631. 10.1093/ps/76.12.1627 9438273

[6] Gosler AG, Barnett PR, Reynolds SJ (2000) Inheritance and variation in eggshell patterning in the great tit *Parus major* . Proceedings of the Royal Society of London B 267:2469–2473. 10.1098/rspb.2000.1307 PMC1690839 11197121

[7] Wang Z, Qu L, Yao J, Yang X, Li G, et al (2013) An *EAV-HP* insertion in 5′ flanking region of *SLCO1B3* causes blue eggshell in the chicken. PLoS Genetics 9:e1003183. 23359636 10.1371/journal.pgen.1003183 PMC3554524

[8] Collias EC (1984) Egg measurements and coloration throughout life in the village weaverbird, *Ploceus cucullatus*. In: Ledger Jeditor. Proceedings of the Fifth Pan-African Ornithological Congress (1980). Johannesburg: South African Ornithological Society. pp. 461–475.

[9] Collias EC (1993) Inheritance of egg-color polymorphism in the village weaver (*Ploceus cucullatus*). Auk 110:683–692.

[10] Dearborn DC, Hanley D, Ballantine K, Cullum J, Reeder DM (2012) Eggshell colour is more strongly affected by maternal identity than by dietary antioxidants in a captive poultry system. Functional Ecology 26:912–920.

[11] Wheelwright NT, Graff ES, Norris DR (2012) Relative consistency in size, shape, and coloration of savannah sparrow eggs within and between breeding seasons. Condor 114:412–420.

[12] Duval C, Cassey P, Miksik I, Reynolds SJ, Spencer KA (2013) Condition-dependent strategies of eggshell pigmentation: an experimental study of Japanese quail (Coturnix coturnix japonica). Journal of Experimental Biology 216:700–708. 23125343 10.1242/jeb.077370

[13] Lahti DC (2005) Evolution of bird eggs in the absence of cuckoo parasitism. Proceedings of the National Academy of Sciences of the U S A 102:18057–18062. 10.1073/pnas.0508930102 PMC1312405 16326805

[14] Hill GE, McGraw KJ, **editors** (2006) Bird Coloration, vol. I: Mechanisms and Measurements. Cambridge, MA: Harvard University Press.

[15] Gosler AG, Higham JP, Reynolds SJ (2005) Why are birds' eggs speckled? Ecology Letters 8:1105–1113.

[16] Moreno J, Lobato E, Morales J, Merino S, Tomas G, et al (2006) Experimental evidence that egg color indicates female condition at laying in a songbird. Behavioral Ecology 17:651–655.

[17] Lahti DC (2008) Population differentiation and rapid evolution of egg color in accordance with solar radiation. Auk 125:796–802.

[18] Cassey P, Maurer G, Lovell PG, Hanley D (2011) Conspicuous eggs and colourful hypotheses: testing the role of multiple influences on avian eggshell appearance. Avian Biology Research 4:185–195.

[19] Jagannath A, Shore RF, Walker LA, Ferns PN, Gosler AG (2007) Eggshell pigmentation indicates pesticide contamination. Journal of Applied Ecology doi:–10.1111/j.1365–2664.2007.01386.x.

[20] Hanley D, Doucet SM (2012) Does environmental contamination influence egg coloration? A long-term study in herring gulls. Journal of Applied Ecology 49:1055–1063.

[21] Morales J, Velando A, Torres R (2011) Biliverdin-based egg coloration is enhanced by carotenoid supplementation. Behavioral Ecology and Sociobiology 65:197–203.

[22] Butler MW, McGraw KJ (2013) Eggshell coloration reflects both yolk characteristics and dietary carotenoid history of female mallards. Functional Ecology 27:1176–1185.

[23] Butcher GD, Miles RD (1995) Factors causing poor pigmentation of brown-shelled eggs. Gainesville, FL: Veterinary Medicine-Large Animal Clinical Sciences Department, Florida Cooperative Extension Service, Institute of Food and Agricultural Sciences, University of Florida. VM94 VM94. 4 p.

[24] Siefferman L, Navara KJ, Hill GE (2006) Egg coloration is correlated with female condition in eastern bluebirds (*Sialia sialis*). Behavioral Ecology and Sociobiology 59:651–656. 10.1007/s00265-007-0416-0 PMC2719904 19655039

[25] Arendt WJ (2004) A quarter century of variation in color and allometric characteristics of eggs from a rain forest population of the pearly-eyed thrasher (*Margarops fuscatus*). Caribbean Journal of Science 40:204–217.

[26] Avilés JM, Stokke BG, Moksnes A, Røskaft E, Møller AP (2007) Environmental conditions influence egg color of reed warblers *Acrocephalus scirpaceus* and their parasite, the common cuckoo *Cuculus canorus* . Behavioral Ecology and Sociobiology 61:475–485.

[27] McCormack JE, Berg EC (2010) Small-scale divergence in egg color along an elevation gradient in the Mexican jay (*Aphelocoma ultramarina*): a condition-dependent response? Auk 127:35–43.

[28] Honza M, Procházka P, Požgayová M (2012) Do weather conditions affect the colouration of great reed warbler Acrocephalus arundinaceus eggs? Folia Zoologica 61:219–224.

[29] Walters M (1994) Birds' Eggs. New York: Dorling Kindersley.

[30] Moreno J, Lobato E, Morales J (2011) Eggshell blue-green colouration fades immediately after oviposition: a cautionary note about measuring natural egg colours. Ornis Fennica 88:51–56.

[31] Pohland G, Mullen P (2006) Preservation agents influence UV-coloration of plumage in museum bird skins. Journal of Ornithology 147:464–467.

[32] Doucet SM, Hill GE (2009) Do museum specimens accurately represent wild birds? A case study of carotenoid, melanin, and structural colours in long-tailed manakins *Chiroxiphia linearis* . Journal of Avian Biology 40:146–156.

[33] Cassey P, Maurer G, Duval C, Ewen JG, Hauber ME (2010) Impact of time since collection on avian eggshell color: a comparison of museum and fresh egg specimens. Behavioral Ecology and Sociobiology 64:1711–1720.

[34] Cassey P, Hauber ME, Maurer G, Ewen JG (2012) Sources of variation in reflectance spectrophotometric data: a quantitative analysis using avian eggshell colours. Methods in Ecology and Evolution 3:450–456.

[35] Yoshizumi K, Crews PC (2003) Characteritics of fading of wool cloth dyed with selected natural dyestuffs on the basis of solar radiant energy. Dyes and Pigments 58:197–204.

[36] Brown BE (1997) Coral bleaching: causes and consequences. Coral Reefs 16:S129–S138.

[37] Hanley D, Stoddard MC, Cassey P, Brennan PLR (2013) Eggshell conspicuousness in ground nesting birds: do conspicuous eggshells signal nest location to conspecifics? Avian Biology Research 6:147–156.

[38] **Heirwegh** KPM, Brown SB, editors (1982) Bilirubin. Boca Raton, FL: CRC Press.

[39] Moan J, Berg K (2008) The photodegradation of porphyrins in cells can be used to estimate the lifetime of singlet oxygen. Photochemistry and Photobiology 53:549–553. 10.1111/j.1751-1097.1991.tb03669.x 1830395

[40] Cato PS (1986) Guidelines for managing bird collections (Museology, no. 7). Lubbock, TX: Texas Tech Press.

[41] Romanoff AL, Romanoff AJ (1949) The Avian Egg. New York: John Wiley & Sons, Inc.

[42] Craig AJFK (2010) Family Ploceidae (Weavers). In: del Hoyo J, Elliott A, Christie DAeditors. Handbook of the Birds of the World. Barcelona: Lynx Edicions. pp. 74–197.

[43] Lahti DC (2013) The sociality of nesting in Rüppell's weaver *Ploceus galbula* and the lesser masked weaver *Ploceus intermedius* in an Ethiopian acacia woodland. Ostrich 84:235–238.

[44] Judd DB, MacAdam DL, Wyszecki G, Budde HW, Condit HR, et al (1964) Spectral distribution of typical daylight as a function of correlated color temperature. Journal of the Optical Society of America 54:1031–1040.

[45] Gomez D (2006) AVICOL, a program to analyse spectrometric data. v6 ed: Free executable available: http://sites.google.com/site/avicolprogram/or from the author at dodogomez@yahoo.fr.

